# Association of physical fitness with visceral fat status and metabolic dysfunction-associated fatty liver disease in individuals with spinal cord injury using manual wheelchair in Korea

**DOI:** 10.1016/j.pmedr.2025.103182

**Published:** 2025-07-17

**Authors:** Minjun Kim, Inhwan Lee

**Affiliations:** a Research Institute of Future Convergence, Changwon National University, Changwon, Republic of Korea; b Department of Smart and Healthcare, Changwon National University, Changwon, Republic of Korea

**Keywords:** Spinal cord injury, Physical fitness, Visceral fat, Fatty liver, Chronic disease

## Abstract

**Objective:**

This study aimed to investigate the association of physical fitness with visceral fat status and metabolic dysfunction-associated fatty liver disease (MAFLD) in individuals with spinal cord injury (SCI) in Korea.

**Methods:**

This cross-sectional study included 113 individuals with SCI (aged ≥40 years; women, 19.5 %) who were receiving care at the disability welfare facilities in G and C Provinces. Data were collected from October 2022 to August 2024. The new visceral adiposity index was used to evaluate visceral fat status. MAFLD was assessed using fatty liver index (FLI), type 2 diabetes, body mass index, and metabolic risk factors. Physical fitness was measured by muscular strength, muscular endurance, flexibility, and cardiorespiratory endurance. After adjusting for age and sex, the participants were grouped into high, middle, and low fitness groups. The odds ratio (OR) and 95 % confidence interval (CI) for abnormal visceral adiposity and MAFLD, according to the fitness level, was calculated using binary logistic regression analysis.

**Results:**

As the physical fitness level was decreasing, a significant linear trend toward increasing new visceral adiposity index (*p* < 0.01) and FLI (p < 0.01) scores were observed. The low fitness group exhibited a higher OR for abnormal visceral adiposity (OR = 3.64, 95 % CI = 1.28–10.37) and MAFLD (OR = 4.39, 95 % CI = 1.48–13.03) than the high fitness group (OR = 1.00).

**Conclusion:**

The findings of this study suggest that improving physical fitness through various regular exercise range may be influential in maintaining an appropriate visceral fat status and preventing MAFLD in individuals with SCI.

## Highlights

• Flexibility was lower with abnormal visceral fat than with normal visceral fat.
• Low fitness group had higher risk of abnormal visceral fat than high fitness group.
• They also had higher metabolic dysfunction-associated fatty liver disease risk.

## 1 Introduction

Recently, metabolic dysfunction-associated fatty liver disease (MAFLD) was defined as a condition addressing several limitations of the traditional concept of non-alcoholic fatty liver disease (NAFLD), including its heterogeneous clinical characteristics and ambiguity surrounding alcohol consumption criteria (Eslam et al., 2020a, Eslam et al., 2020b; Stefano et al., 2023). MAFLD diagnosis requires hepatic steatosis and metabolic risk factors (obesity, type 2 diabetes, waist circumference, blood pressure, blood lipids, and fasting glucose) being present, reflecting a more precise reflection of an individual's overall metabolic health, which holds significant clinical relevance (Eslam et al., 2020a, Eslam et al., 2020b). MAFLD, increasingly regarded as a liver condition and key health indicator, relates closely to metabolic dysfunction, and justifies the increasing attention for accurate understanding and management (Eslam et al., 2020a, Eslam et al., 2020b).

The 2023 South Korea disability prevalence rate was approximately 5.1 % (2.63 million people), including 44.4 % (1.17 million) individuals with physical disabilities (Ministry of Health and Welfare, 2023). These individuals are more vulnerable to chronic diseases than the general population (Dixon-Ibarra and Horner-Johnson, 2014). Particularly, individuals with spinal cord injury (SCI) may report lower physical activity levels than the general population, experience higher obesity and metabolic syndrome prevalence, and show associated increased fatty liver disease risk and impaired liver function (Gorgey et al., 2014; Goodus and McTigue, 2020). Physical activity and fitness enhancement, critical factors in improving fatty liver in the general population, play a positive role in MAFLD prevention (Lee et al., 2023; Keating et al., 2023). Thus, increasing physical activity and fitness may address MAFLD in individuals with SCI, particularly those using wheelchair and engaging in upper-body activities. However, research on this topic remains lacking.

The pathological mechanisms underlying hepatic fat accumulation include hormonal and cytokine imbalances, chronic inflammation, cellular stress, and insulin resistance (Kuchay et al., 2020). In individuals with disabilities, increased visceral fat from reduced physical activity contributes to pathological deterioration. Consequently, several studies investigated physical fitness role in mitigating these adverse fat-related outcomes (Højberg et al., 2022, Silva et al., 2017). Among individuals with cognitive disabilities, cardiorespiratory and muscular endurance are independent predictors of obesity-related indicators, including body fat percentage and waist-to-hip ratio (Gawlik et al., 2016; Jeong and Chun, 2021). Likewise, in patients with multiple sclerosis, obesity indicators significantly correlated with walking speed, dynamic balance, and gait stability (Kalron, 2017). Thus, in individuals with disabilities, physical fitness enhancement relates closely with visceral fat reduction and healthy body composition maintenance, irrespective of disability type. Consequently, by improving physical fitness in individuals with SCI, appropriate visceral fat levels may be positively maintained; however, related prior research is limited.

Furthermore, enhancing physical fitness in the general population positively impact several chronic conditions, including metabolic syndrome, diabetes, kidney disease, and MAFLD, which are strongly associated with metabolic disorders (Chang et al., 2023; Kunutsor et al., 2023; Słomko et al., 2021). Schnurr et al. (2022), by identifying muscle strength and gait function as risk factors for MAFLD, support these, and this was further emphasized by Noda et al. (2022). Despite the higher vulnerability of individuals with SCI to fatty liver disease owing to functional impairments, no previous studies have verified the role of fitness in MAFLD in this population. Therefore, this study investigated physical fitness role in the visceral fat state and MAFLD among individuals with SCI. We hypothesized that higher physical fitness levels would be associated with reduced visceral adiposity and lower MAFLD prevalence in individuals with SCI.

## 2 Methods

### 2.1 Study participants

Since reported MAFLD prevalence increases markedly after age 40 (Wong and Cheung, 2022), this study included individuals with chronic SCI using manual wheelchairs and diagnosed with SCI by a physician ≤1 year post-injury and before study commencement. Eligible participants, were aged ≥40 years, from disability welfare facilities in G and C Provinces, registered as persons with severe spinal cord disability under the Korean national disability system with comparable impairment levels of grade 1 or 2 as previously classified, and were full-time manual wheelchair users (Kim et al., 2023). A priori sample size calculated using G*Power (version 3.1.9.4), assumed 0.15 effect size, 0.05 significance level (α), and 0.80 power (1–β), for three groups, was 98. After accounting for a 20 % dropout rate, 120 participants were recruited. Five and two participants were excluded during data collection because of no physical fitness assessments and incomplete blood analysis data, respectively, leaving 113 participants' data, collected from October 2022 to August 2024, for the final analysis. All participants received explanations on the study details, and provided written informed consent before participating. The Institutional Review Board of Changwon National University approved the study protocol (7001066-202401-HR-009).

### 2.2 Body composition and blood pressure

Heights (in cm) of participants lying flat on a mat, were measured using a tape measure, from the soles of the feet to crown of the head. Weights of participants were calculated by subtracting the wheelchair's weight, measured using a wheelchair scale (AD-6105NP, AND, Seoul, Korea) with the participant seated in it, from the total recorded. Body fat percentage and appendicular muscle mass were measured using a bioelectrical impedance analysis-based body composition analyzer (S10, Inbody, Seoul, Korea) with the participant lying flat on a bed. Body mass index (BMI) and appendicular skeletal muscle mass (ASM) index were calculated using the formulae weight (kg)/height (m^2^) and appendicular muscle mass (kg)/height (m^2^), respectively. The average blood pressure (BP) from two measurements after adequate rest using an automatic BP monitor (HEM-7156, Omron, Kyoto, Japan), included systolic BP (SBP) and diastolic BP (DBP). Mean BP (MBP) was calculated using the formula DBP + 1/3(SBP-DBP) (Sesso et al., 2000). Waist circumference was measured in cm using a tape measure at the midpoint between the iliac crest and last rib.

### 2.3 Visceral fat status and MAFLD

Visceral fat was assessed using the new visceral adiposity index (NVAI), validated in Korean populations (Oh et al., 2018). NVAI incorporates age, waist circumference, MBP, triglycerides, and high-density lipoprotein cholesterol (HDL-C). Participants were classified into normal (NVAI<0.95) and abnormal (NVAI ≥0.95) adiposity groups, using the following estimation formulae (Sun et al., 2023):

$$\mathrm{Men}:\text{NVAI}=1/\left[1+\exp.\left\{-\left(-21.858+\left(0.099^{*}\;\mathrm{age}\right)+\left(0.10^{*}\;\text{waist circumference}\right)+\left(0.12^{*}\;\mathrm{MBP}\right)+\left(0.006^{*}\;\text{triglyceride}\right)+\left(-0.077^{*}\;\mathrm{HDL}-C\right)\right)\right\}\right]$$

$$\text{Women}:\text{NVAI}=1/1\left[1+\exp.\left\{-\left(-18.765+\left(0.058^{*}\;\mathrm{age}\right)+\left(0.14^{*}\;\text{waist circumference}\right)+\left(0.057^{*}\;\mathrm{MBP}\right)+\left(0.004^{*}\;\text{triglyceride}\right)+\left(-0.057^{*}\;\mathrm{HDL}-C\right)\right)\right\}\right]$$

MAFLD was defined as a combination of fatty liver index (FLI) of ≥60 with either type 2 diabetes or BMI ≥ 23 kg/m^2^, as previously reported. Alternatively, for individuals with BMI < 23 kg/m^2^, MAFLD was based on at least two of the following: waist circumference (men≥90 cm and women≥80 cm); BP (SBP ≥ 130 mmHg or DBP ≥ 85 mmHg); triglyceride (≥150 mg/dL); HDL-C (men <40 mg/dL and women<50 mg/dL); or prediabetes (fasting blood glucose [FBG] 100–125 mg/dL) (Eslam et al., 2020a, Eslam et al., 2020b). FLI was calculated using the formula (Bedogni et al., 2006):

$$\mathrm{FLI}=1/{\left(1+\exp.\left(-x\right)\right)}^{*}\;100,$$

$$x=0.953^{*}\;\log_{e}\left(\text{triglyceride}\right)+0.139^{*}\;\mathrm{BMI}+0.718^{*}\;\log_{e}\left(\text{gamma glutamyl}-\text{transferase}\;\left[\mathrm{GGT}\right]\right)+0.053^{*}\;\text{waist circumference}-15.745$$

### 2.4 Physical fitness assessment and group classification

Physical fitness evaluation items for individuals with SCI, including muscle strength, muscular endurance, flexibility, and cardiorespiratory endurance, by the Korea Disability Fitness Certification Center, were assessed (Korea Paralympic Committee, 2018).

Muscle strength was evaluated by measuring grip strength twice per hand, and relative grip strength, using the formula: maximum grip strength (kg)/BMI (kg/m^2^). Muscular endurance, using an arm curl test, was assessed for 2 min in the dominant arm, and 4 and 2 kg weights for men and women, respectively. Flexibility, measured as the maximum reach of both hands by a back scratch test, was the highest value recorded. Cardiorespiratory endurance was the total distance covered recorded when participants travelled back and forth over a 20 m distance in a wheelchair for 5 min (Korea Paralympic Committee, 2018).

The Korea Disability Fitness Certification Center had no official scoring system to comprehensively quantify overall fitness, despite available standardized fitness evaluation items for individuals with SCIn (Korea Paralympic Committee, 2018). Accordingly, following previous research, we employed a stratified tertile classification approach using actual performance data (Atencio-Osorio et al., 2022). Participants were first stratified by sex and age group (≤65 or > 65 years). Within each stratum, *Z*-scores for four physical fitness variables were summed to create a composite Z-score. Participants were then ranked and grouped into fitness tertiles: high (top 33 %), middle (middle 33 %), and low (bottom 33 %) (Atencio-Osorio et al., 2022).

### 2.5 Blood variable analysis

Approximately 70 μL of whole blood from the participant's non-dominant hand thumb using a lancet was collected after at least 10-h fasting, placed in a Biochemistry Test 9 cartridge, and spectrophotometric analysis was performed using a blood lipid analyzer (Exdia PT10, Precision Biosensor, Daejeon, Korea). The analysis included HDL-C, triglyceride, GGT, and FBG measurements.

### 2.6 Covariates

Covariates included disability characteristics (duration and injury location; sociodemographic factors (income, education level, and marital status); and health-related factors (smoking, heavy alcohol consumption, physical inactivity, and menopause). Injury locations were cervical, thoracic, lumbar, or transverse myelitis. Education levels were high school graduate or lower and associate degree or higher. Smoking status was either currently smoking or smoked at least 100 cigarettes in the past (CDC, 1994). Heavy alcohol consumption was drinking ≥15 (men) or ≥ 8 (women) drinks per week (Xi et al., 2017). Physical inactivity was engaging in <150 min of physical activity per week, irrespective of intensity (Bull et al., 2020).

### 2.7 Data analysis

Continuous variables are presented as means and standard deviations, and categorical variables as proportions, per group. Independent samples *t*-test was used to examine differences in means according to sex, visceral fat status, and MAFLD. Polynomial contrasts in one-way analysis of variance and linear-by-linear chi-squared tests were used to verify linear trends of measurements by fitness level. Binary logistic regression analysis was performed to calculate odds ratios (ORs) for abnormal visceral fat and MAFLD by fitness level, with 95 % confidence intervals (CIs). Significant hypothesis testing level was set at α = 0.05, and all statistical analyses were conducted using SPSS-PC version 23 (IBM corp., Armonk, NY, USA).

## 3 Results

### 3.1 Comparison of measured characteristics by sex

This study included 113 individuals with SCI (91 men, 22 women) classified as having severe disabilities according to the national disability grading system, and were all manual wheelchair users (Ministry of Health and Welfare, 2023). Men had significantly higher heights (*p* < 0.01), weights (*p* < 0.01), ASM index (*p* < 0.01), body fat percentage (*p* < 0.01), waist circumference (*p* < 0.01), smoking (*p* < 0.01), NVAI (p < 0.01), muscular endurance (*p* < 0.05), muscle strength (*p* < 0.01), and cardiorespiratory endurance (*p* < 0.01) than women (Table 1).

**Table 1:** Distributions of measured parameters by sex among individuals with spinal cord injury in Korea, October 2022 to August 2024.

Variables	Total (*n* = 113)	Men (*n* = 91)	Women (*n* = 22)	*P* value
	n (%) / mean (SD)			
Age (years)	57.4 (8.0)	56.9 (7.9)	59.3 (8.1)	0.21
Duration of injury (years)	24.7 (11.1)	24.5 (10.9)	25.7 (11.8)	0.66
Location of injury				0.05
Cervical	27 (23.9)	23 (25.3)	4 (18.2)	
Thoracic	60 (53.1)	48 (52.7)	12 (54.5)	
Lumbar	19 (16.8)	17 (18.7)	2 (9.1)	
Myelitis	7 (6.2)	3 (3.3)	4 (18.2)	
Body composition				
Height (cm)	165.7 (7.8)	168.0 (6.5)	156.5 (5.7)	<0.01
Weight (kg)	67.1 (11.9)	69.3 (11.1)	58.4 (11.3)	<0.01
BMI (kg/m^2^)	24.5 (4.0)	24.6 (3.9)	23.8 (4.1)	0.36
ASM index (kg/m^2^)	6.4 (1.2)	6.7 (1.1)	5.2 (1.1)	<0.01
Body fat (%)	38.7 (8.0)	37.5 (7.7)	43.5 (7.2)	<0.01
Waist circumference (cm)	92.5 (11.5)	93.9 (11.0)	86.7 (12.2)	<0.01
Socio-economic status				
Income (10,000 won/month)	313.2 ± 242.0	311.2 ± 233.7	321.5 ± 279.3	0.86
Education				0.87
Lower than high school	91 (80.5)	73 (80.2)	18 (81.8)	
Over than college	22 (19.5)	18 (19.8)	4 (18.2)	
Marital status				0.35
Married	78 (69.0)	61 (67.0)	17 (77.3)	
Widowed/divorced/unmarried	35 (31.0)	30 (33.0)	5 (22.7)	
Health related factors				
Smoking	70 (61.9)	67 (73.6)	3 (13.6)	<0.01
Heavy alcohol	15 (13.3)	12 (13.2)	3 (13.6)	0.96
Physical inactivity	27 (23.9)	22 (24.2)	5 (22.7)	0.89
Diabetes	22 (19.5)	20 (22.0)	2 (9.1)	0.17
Menopause	18 (15.9)	0 (0.0)	18 (81.8)	<0.01
Metabolic syndrome components	2.9 (1.3)	3.0 (1.2)	2.7 (1.4)	0.33
MBP (mmHg)	95.9 (13.1)	95.6 (13.4)	97.1 (12.1)	0.64
Laboratory parameters				
HDL-C (mg/dL)	46.5 (10.7)	45.8 (11.1)	49.4 (8.5)	0.16
Triglyceride (mg/dL)	164.6 (77.9)	159.8 (79.1)	184.2 (71.0)	0.19
GGT (IU/L)	31.2 (27.3)	32.0 (28.8)	27.7 (20.1)	0.51
FBG (mg/dL)	111.1 (24.4)	112.4 (25.7)	105.5 (17.8)	0.23
NVAI score	0.70 (0.31)	0.75 (0.29)	0.50 (0.33)	<0.01
FLI score	43.5 (26.4)	44.8 (25.8)	37.9 (28.6)	0.27
Physical fitness parameters				
Muscular endurance (rep/2 min)	88.4 (27.1)	90.9 (26.8)	77.8 (26.5)	<0.05
Muscular strength (kg/BMI)	1.46 (0.53)	1.57 (0.50)	1.01 (0.38)	<0.01
Flexibility (cm)	−19.16 (11.31)	−20.1 (11.1)	−15.5 (11.5)	0.09
Cardiorespiratory endurance (m/5 min)	414.2 (123.2)	436.3 (112.9)	322.8 (124.3)	<0.01

**Note:***P*-values for comparing between sexes were obtained using chi-squared or t-test. As at 9 July 2025, 10,000 won was equivalent to approximately 7.30 US dollars.

**Abbreviations:** BMI: body mass index, ASM: appendicular skeletal muscle mass, MBP: mean blood pressure, HDL-C: high density lipoprotein cholesterol, GGT: gamma glutamyl-transferase, FBG: fasting blood glucose, NVAI: new visceral adiposity index, FLI: fatty liver index.

### 3.2 Comparison of characteristics by visceral fat status and MAFLD

The elevated visceral fat group (NVAI≥0.95) had significantly higher age (*p* < 0.01), weight (p < 0.01), BMI (p < 0.01), ASM index (p < 0.01), body fat percentage (*p* < 0.05), waist circumference (p < 0.01), diabetes (p < 0.05), number of metabolic syndrome components (*p* < 0.01), MBP (*p* < 0.01), and triglyceride (p < 0.05) than the NVAI<0.95 group. Conversely, the elevated visceral fat group had fewer women (*p* = 0.05), and significantly lower HDL-C (*p* < 0.01) value and flexibility (*p* < 0.05) (Table 2).

**Table 2:** Comparison of measured parameters by visceral adiposity and metabolic dysfunction-associated fatty liver disease status among individuals with spinal cord injury in Korea, October 2022 to August 2024.

Variables	Visceral adiposity status			MAFLD		
	NVAI<0.95 (*n* = 78)	NVAI ≥0.95 (*n* = 35)	P value	Normal (*n* = 82)	Case (*n* = 31)	P value
	n (%) / mean (SD)			n (%) / mean (SD)		
NVAI score	0.58 (0.30)	0.98 (0.01)	<0.01	0.63 (0.32)	0.91 (0.16)	<0.01
FLI score	35.4 (23.6)	61.6 (23.4)	<0.01	30.2 (16.4)	78.5 (11.4)	<0.01
Age (years)	55.9 (7.8)	60.5 (7.5)	<0.01	57.1 (7.5)	58.1 (9.3)	0.52
Duration of injury (years)	25.3 (11.2)	23.6 (10.8)	0.45	26.2 (10.6)	20.9 (11.4)	<0.05
Women	19 (24.4)	3 (8.6)	0.05	16 (19.5)	6 (19.4)	0.99
Location of injury			0.19			0.28
Cervical	18 (23.1)	9 (25.7)		16 (19.5)	11 (35.5)	
Thoracic	38 (48.7)	22 (62.9)		45 (54.9)	15 (48.4)	
Lumbar	17 (21.8)	2 (5.7)		16 (19.5)	3 (9.6)	
Myelitis	5 (6.4)	2 (5.7)		5 (6.1)	2 (6.5)	
Body composition parameters						
Height (cm)	165.8 (8.5)	165.6 (6.0)	0.93	165.3 (8.0)	166.9 (7.3)	0.32
Weight (kg)	64.2 (11.2)	73.6 (11.0)	<0.01	62.5 (8.7)	79.4 (10.5)	<0.01
BMI (kg/m^2^)	23.4 (3.5)	26.8 (3.9)	<0.01	22.9 (2.9)	28.7 (3.4)	<0.01
ASM index (kg/m^2^)	6.1 (1.2)	6.9 (1.1)	<0.01	6.0 (1.1)	7.3 (1.2)	<0.01
Body fat (%)	37.7 (8.4)	40.9 (6.5)	<0.05	37.3 (7.7)	42.3 (7.5)	<0.01
Waist circumference (cm)	89.1 (10.5)	100.3 (10.0)	<0.01	88.1 (8.6)	104.4 (9.9)	<0.01
Socio-economic status						
Income (10,000 won/month)	321.2 ± 256.6	295.3 ± 208.1	0.60	335.0 ± 250.3	255.5 ± 211.2	0.12
Education			0.35			0.28
Lower than high school	61 (78.2)	30 (85.7)		64 (78.0)	27 (87.1)	
Over than college	17 (21.8)	5 (14.3)		18 (22.0)	4 (12.9)	
Marital status			0.19			0.62
Married	53 (67.9)	25 (71.4)		58 (70.7)	20 (64.5)	
Widowed/divorced/unmarried	25 (32.1)	10 (28.6)		24 (29.3)	11 (35.5)	
Health related factors						
Smoking	45 (57.7)	25 (71.4)	0.16	51 (62.2)	19 (61.3)	0.93
Heavy alcohol	10 (12.8)	5 (14.3)	0.83	12 (14.6)	3 (9.7)	0.49
Physical inactivity	21 (26.9)	6 (17.1)	0.26	19 (23.2)	8 (25.8)	0.77
Diabetes	11 (14.1)	11 (31.4)	<0.05	16 (19.5)	6 (19.4)	0.99
Menopause	15 (19.2)	3 (8.6)	0.15	12 (14.6)	6 (19.4)	0.54
Metabolic syndrome components	2.4 (1.1)	4.0 (0.9)	<0.01	2.5 (1.1)	4.0 (0.9)	<0.01
MBP (mmHg)	91.3 (10.9)	106.2 (11.8)	<0.01	95.0 (13.2)	98.3 (12.7)	0.24
Blood markers						
HDL-C (mg/dL)	48.7 (11.2)	41.7 (7.5)	<0.01	47.7 (10.6)	43.4 (10.4)	0.06
Triglyceride (mg/dL)	154.7 (76.2)	186.6 (78.1)	<0.05	149.6 (74.8)	204.2 (72.7)	<0.01
GGT (IU/L)	30.6 (30.8)	32.5 (17.5)	0.73	23.8 (9.9)	50.7 (44.4)	<0.01
FBG (mg/dL)	108.8 (23.9)	116.1 (25.2)	0.14	111.5 (27.0)	109.9 (15.9)	0.76
Physical fitness parameters						
Muscular endurance (rep/2 min)	88.8 (26.2)	87.5 (29.6)	0.82	90.7 (24.7)	82.3 (32.5)	0.14
Muscular strength (kg/BMI)	1.52 (0.55)	1.34 (0.46)	0.10	1.56 (0.47)	1.21 (0.60)	<0.01
Flexibility (cm)	−17.5 (12.1)	−22.8 (8.5)	<0.05	−17.6 (11.9)	−23.3 (8.3)	<0.05
Cardiorespiratory endurance (m/5 min)	421.9 (111.4)	397.0 (146.6)	0.32	433.6 (101.6)	362.9 (158.2)	<0.01

Note: P-values comparing visceral fat status and presence of metabolic dysfunction-associated fatty liver disease were obtained using chi-squared or t-test. As at 9 July 2025, 10,000 won was equivalent to approximately 7.30 US dollars.

Abbreviations: NVAI: new visceral adiposity index, MAFLD: metabolic dysfunction associated fatty liver disease, FLI: fatty liver index, BMI: body mass index, ASM: appendicular skeletal muscle mass, MBP: mean blood pressure, HDL-C: high density lipoprotein cholesterol, GGT: gamma glutamyl-transferase, FBG: fasting blood glucose.

Those with MAFLD had significantly higher weights (p < 0.01), BMI (p < 0.01), ASM index (p < 0.01), body fat percentage (p < 0.01), waist circumference (p < 0.01), number of metabolic syndrome components (p < 0.01), triglyceride (p < 0.01), and GGT (p < 0.01) than the non-MAFLD group. However, the MAFLD group showed significantly lower disability duration (p < 0.05), muscle strength (p < 0.01), flexibility (p < 0.05), and cardiorespiratory endurance (p < 0.01) (Table 2).

### 3.3 Comparison of characteristics by physical fitness levels

There was a significant linear trend, with lower fitness levels associated with higher age (p < 0.01), weight (p < 0.01), BMI (p < 0.01), body fat percentage (p < 0.01), waist circumference (p < 0.01), number of metabolic syndrome components (p < 0.05), NVAI score (p < 0.01), and FLI score (p < 0.01) (Table 3).

**Table 3:** Comparison of measured parameters by physical fitness levels among individuals with spinal cord injury in Korea, October 2022 to August 2024.

Variables	High fitness (*n* = 37)	Middle fitness (*n* = 39)	Low fitness (n = 37)	P for linear trends
	n (%) / mean (SD)			
Physical fitness parameters				
Physical fitness *Z*-score	0.80 (0.33)	0.00 (0.20)	−0.80 (0.44)	<0.01
Muscular endurance (rep/2 min)	109.2 (20.0)	92.2 (19.6)	63.5 (19.6)	<0.01
Muscular strength (kg/BMI)	1.85 (0.43)	1.43 (0.41)	1.11 (0.46)	<0.01
Flexibility (cm)	−9.9 (11.5)	−21.6 (8.1)	−25.9 (7.5)	<0.01
Cardiorespiratory endurance (m/5 min)	503.0 (75.4)	430.4 (90.6)	308.4 (113.9)	<0.01
Socio-demographic status				
Age (years)	52.4 (7.5)	59.1 (6.3)	60.5 (7.9)	<0.01
Duration of injury (years)	26.1 (11.4)	25.3 (10.7)	22.8 (11.0)	0.19
Women, n (%)	7 (18.9)	8 (20.5)	7 (18.9)	0.98
Location of injury, n (%)				0.25
Cervical	7 (18.9)	4 (10.3)	16 (43.2)	
Thoracic	23 (62.2)	23 (59.0)	14 (10.8)	
Lumbar	5 (13.5)	10 (25.6)	4 (10.8)	
Myelitis	2 (5.4)	2 (5.1)	7 (6.2)	
Body composition				
Height (cm)	167.2 (7.9)	165.2 (8.1)	164.9 (7.3)	0.21
Weight (kg)	62.8 (11.3)	67.3 (11.1)	71.4 (12.1)	<0.01
BMI (kg/m^2^)	22.4 (3.2)	24.7 (3.7)	26.3 (4.1)	<0.01
ASM index (kg/m^2^)	6.2 (1.3)	6.2 (1.3)	6.7 (1.1)	0.15
Body fat (%)	34.4 (7.6)	40.5 (6.9)	41.1 (7.8)	<0.01
Waist circumference (cm)	87.1 (9.9)	92.5 (8.6)	98.0 (13.3)	<0.01
Socio-economic status				
Income (10,000won/month)	361.0 ± 294.1	317.6 ± 226.1	260.8 ± 191.1	0.08
Education, n (%)				0.24
Lower than high school	27 (73.0)	33 (84.6)	31 (83.8)	
Over than college	10 (27.0)	6 (15.4)	6 (16.2)	
Marital status, n (%)				0.27
Married	25 (67.6)	26 (66.7)	27 (73.0)	
Widowed/divorced/unmarried	12 (32.4)	13 (33.3)	10 (27.0)	
Health related factors				
Smoking, n (%)	25 (67.6)	20 (51.3)	25 (67.6)	0.99
Heavy alcohol, n (%)	7 (18.9)	4 (10.3)	4 (10.8)	0.31
Physical inactivity (n, %)	10 (27.0)	11 (28.2)	6 (16.2)	0.27
Diabetes, n (%)	5 (13.5)	8 (20.5)	9 (24.3)	0.24
Menopause, n (%)	3 (8.1)	8 (20.5)	7 (18.9)	0.21
Metabolic syndrome components	2.6 (1.3)	2.9 (1.2)	3.2 (1.2)	<0.05
MBP (mmHg)	94.8 (12.2)	97.7 (12.3)	95.2 (14.8)	0.88
Laboratory parameters				
HDL-C (mg/dL)	45.8 (9.1)	47.8 (9.4)	45.9 (13.3)	0.96
Triglyceride (mg/dL)	164.9 (83.3)	158.9 (74.4)	170.2 (77.5)	0.77
GGT (IU/L)	28.3 (18.7)	29.7 (18.5)	35.7 (39.7)	0.25
FBG (mg/dL)	110.4 (24.7)	113.1 (27.9)	109.6 (20.4)	0.90
NVAI score	0.58 (0.35)	0.76 (0.26)	0.77 (0.30)	<0.01
FLI score	31.3 (22.5)	44.1 (23.3)	55.0 (28.2)	<0.01

**Note:** Fitness groups were defined based on composite Z-scores for four physical fitness variables. Participants were stratified by sex and age group (≤65 or > 65 years), and divided into groups by tertiles: high (top 33 %), middle (middle 33 %), and low (bottom 33 %). P-values comparing physical fitness levels were obtained using linear-by-linear chi-square test and polynomial contrasts in one-way analysis of variance. As at 9 July 2025, 10,000 won was equivalent to approximately 7.30 US dollars.

**Abbreviations:** BMI: body mass index, ASM: appendicular skeletal muscle mass, MBP: mean blood pressure, HDL-C: high density lipoprotein cholesterol, GGT: gamma glutamyl-transferase, FBG: fasting blood glucose, NVAI: new visceral adiposity index, FLI: fatty liver index.

### 3.4 Risk of abnormal visceral fat and MAFLD by fitness levels

Table 4 presents the risks of abnormal visceral fat and MAFLD by fitness level. Regarding the visceral fat status, the low fitness group showed a significantly higher risk of abnormal visceral fat (OR = 3.64, 95 % CI = 1.28–10.37) than the high fitness group (OR = 1.00).

**Table 4:** Odds ratios for abnormal visceral adiposity and metabolic dysfunction-associated fatty liver disease by physical fitness levels among individuals with spinal cord injury in Korea, October 2022 to August 2024.

Variable	Model 1	Model 2
	OR (95 % CI)	OR (95 % CI)
NVAI ≥0.95		
High fitness	1.00 (reference)	1.00 (reference)
Middle fitness	1.68 (0.57–4.95)	1.60 (0.50–5.10)
Low fitness	3.64 (1.28–10.37)	2.81 (0.88–9.01)
MAFLD		
High fitness	1.00 (reference)	1.00 (reference)
Middle fitness	1.33 (0.41–4.30)	1.21 (0.33–4.42)
Low fitness	4.39 (1.48–13.03)	3.73 (1.06–13.10)

**Note:** Fitness groups were defined based on composite Z-scores for four physical fitness variables. Participants were stratified by sex and age group (≤65 or > 65 years), and divided into groups by tertile: high (top 33 %), middle (middle 33 %), and low (bottom 33 %). Odds ratio and 95 % confidence interval using binary logistic regression analyses for high visceral fat status and presence of metabolic dysfunction-associated fatty liver disease according to physical fitness levels, with the high fitness level as the reference category. Model 1: Unadjusted. Model 2: Adjusted for age, sex, duration of injury, location of injury, income, education, marital status, smoking, heavy alcohol, physical inactivity, and menopause.

**Abbreviations:** NVAI: new visceral adiposity index, MAFLD: metabolic dysfunction associated fatty liver disease, OR: odds ratio, CI: confidence interval.

Similarly, the low fitness group showed a significantly higher risk of MAFLD (OR = 4.39, 95 % CI = 1.48–13.03) than the high fitness group (OR = 1.00). This result remained significant in model 2, adjusted for age, sex, injury duration, injury location, income, education level, marital status, smoking status, heavy alcohol consumption, physical inactivity, and menopausal status (OR = 3.73, 95 % CI = 1.06–13.10).

## 4 Discussion

This study findings showed that, compared to the elevated visceral fat group with NVAI<0.95 that elevated visceral fat group with NVAI≥0.95 had lower flexibility, whereas those with MAFLD demonstrated reduced muscle strength, flexibility, and cardiorespiratory endurance. Additionally, the low fitness group had a significantly higher risk of elevated visceral fat and MAFLD than the high fitness group, and these associations remained significant even after adjusting for relevant covariates.

Recently, social attention on MAFLD has increased, and visceral fat accumulation reportedly induced pathological progression of fatty liver disease (Kuchay et al., 2020). Additionally, individuals with disabilities have a higher risk of liver diseases than the general population (Dixon-Ibarra and Horner-Johnson, 2014). In particular, individuals with SCI, especially those using wheelchairs, are more vulnerable due to limited lower-body physical activity and associated metabolic changes (Gorgey et al., 2014). In this study, the prevalence rates of abnormal visceral fat and MAFLD of 31.0 % and 27.4 %, respectively, are somewhat higher than the previously reported results. This discrepancy are attributed to the higher prevalence of chronic diseases among individuals with disabilities than that in the general population, and because the study population included individuals with SCI (Jung et al., 2024; Ha et al., 2024).

Physical fitness positively contributes to health outcomes regardless of sex and age (Dhuli et al., 2022; Miller et al., 2016). However, despite significant limitations in physical activity levels and higher risks of abdominal obesity and visceral fat accumulation in individuals with SCI, limited studies have investigated the relationship between physical fitness and obesity-related indicators among them, unlike in the general population, who typically have higher physical activity levels and lower risk of abdominal obesity (Martin Ginis et al., 2021). This study estimated the risk of abnormal visceral fat by fitness levels, and found that the low fitness group had a 3.6-fold higher risk than the high fitness group, which remained approximately 3-fold higher after adjusting for age and sex. These results are similar to those of Wiyanad et al. (2022), who reported a significant correlation between muscular endurance and trunk fat in Asian individuals with SCI, and Hoevenaars et al. (2023), who found improvement in abdominal obesity through increased cardiorespiratory endurance following sufficient physical activity in European individuals with SCI. These results suggest that fitness levels, including muscle strength and muscular endurance in individuals with SCI, are associated with maintaining an appropriate body composition and play a positive role in preventing fatty liver disease development by maintaining normal visceral fat (Kalron, 2017; Rankin et al., 2017).

Additionally, individuals with SCI have a higher prevalence of fatty liver disease than the general population (Eisenberg et al., 2022). In the general population, regular physical activity and high cardiorespiratory fitness are associated with improved liver health, including reduced hepatic fat, liver cell apoptosis, and insulin resistance (Fealy et al., 2012; Kantartzis et al., 2009; Nath et al., 2020). However, despite attempts to investigate the role of fitness in predicting physical function in individuals with SCI, no research has examined the role of fitness in MAFLD. Here, MAFLD risk according to fitness levels was calculated, and the low fitness group had a significantly higher risk of MAFLD than the high fitness group, even after adjusting for covariates. Although our study was cross-sectional, the findings are conceptually supported by Nightingale et al. (2017), an interventional study in which regular upper-body exercise improved hepatic insulin sensitivity in individuals with SCI. Similarly, Clayton-Chubb et al. (2024) found that grip strength and gait function in individuals with mobility impairments may serve as independent predictors of MAFLD. Altogether, our findings and prior evidence suggest that improving fitness through regular exercise in individuals with SCI may contribute to better insulin sensitivity and reduced hepatic fat accumulation. This indicates a potential protective role of physical fitness against MAFLD development, driven by increased visceral fat (Goldsmith et al., 2021; Hoevenaars et al., 2023).

However, this study had some limitations**.** First, the analysis neither distinguished between SCI types or levels (e.g., cervical, thoracic, and lumbar), nor considered SCI completeness or severity. Thus, the findings cannot be generalized to all subgroups of individuals with SCI, and interpretation is limited with respect to specific injury characteristics. Second, this study focused only on the presence or absence of MAFLD and not its subtypes or stages. Future research should examine the association of fitness with different MAFLD stages or phenotypes, in this population. Third, although NVAI was validated for assessing visceral adiposity in the general population, its direct applicability to individuals with SCI is not fully established and measurement bias may have been introduced. Finally, as this was a cross-sectional study, causal inferences between fitness levels and visceral fat or MAFLD development could not be established. Therefore, longitudinal and interventional studies are needed to clarify the causal role of fitness in reducing visceral fat or preventing MAFLD in individuals with SCI. Despite these limitations, this study is the first to examine the association between physical fitness and visceral fat status and MAFLD in individuals with SCI, and offers valuable preliminary evidence to inform future preventive health strategies for this high-risk population.

## 5 Conclusion

Higher fitness levels in individuals with SCI were an independent predictor of abnormal visceral fat and MAFLD risk. Therefore, regular exercise to improve fitness, within feasible limits, can positively influence the maintenance of an appropriate visceral fat status and MAFLD prevention in individuals with SCI.

## CRediT authorship contribution statement

**Minjun Kim:** Writing – review & editing, Writing – original draft, Visualization, Validation, Software, Methodology, Investigation, Formal analysis, Data curation. **Inhwan Lee:** Writing – review & editing, Writing – original draft, Validation, Supervision, Resources, Project administration, Methodology, Funding acquisition, Conceptualization.

## Funding

This work was supported by the Basic Science Research Program through the National Research Foundation of Korea (NRF) funded by the 10.13039/501100002701Ministry of Education (grant number: NRF-2022R1I1A1A01066469).

## Declaration of competing interest

The authors declare that they have no known competing financial interests or personal relationships that could have appeared to influence the work reported in this paper.

## Data Availability

Data will be made available upon reasonable request to the corresponding author immediately following publication to anyone wishing to access the data.
